# Pharmacotherapeutic Options for Managing Neuropathic Pain: A Systematic Review and Meta-Analysis

**DOI:** 10.1155/2021/6656863

**Published:** 2021-04-26

**Authors:** Giulia Di Stefano, Andrea Di Lionardo, Giuseppe Di Pietro, Giorgio Cruccu, Andrea Truini

**Affiliations:** Department of Human Neuroscience, Sapienza University of Rome, Rome, Italy

## Abstract

Despite an increasing number of available therapies, the treatment of neuropathic pain remains a major issue. Systematic reviews and meta-analyses indicate that only a minority of patients with neuropathic pain have an adequate response to pharmacological treatment and that most drugs have dose-limiting side effects. We conducted a systematic review and meta-analysis of randomised controlled trials published in the last five years. We searched for relevant papers within PubMed, EMBASE, the Cochrane Database of Systematic Reviews, and the Clinical Trials database (ClinicalTrials.gov). Two authors independently selected studies for inclusion, data extraction, and bias assessment. We identified 39 randomised controlled trials and included 16 in the meta-analysis. Trial outcomes were generally modest even for first-line drugs such as tricyclic antidepressants, serotonin-noradrenaline reuptake inhibitors, and gabapentinoids. Many drugs acting on new pain targets are currently under development. Clinical data are currently available for sodium channel isoform-specific antagonists, anti-nerve growth factor molecules, and fatty acid amide hydrolase inhibitors.

## 1. Introduction

Despite an increasing number of available therapies, the treatment of neuropathic pain remains a major issue. Systematic reviews and meta-analyses indicate that only a minority of patients with neuropathic pain have an adequate response to pharmacological treatment and that most drugs have dose-limiting side effects [1, 2].

In 2015, the Neuropathic Pain Special Interest Group (NeuPSIG) of the International Association for the Study of Pain (IASP) conducted a systematic review and meta-analysis that included 229 randomised double-blind trials testing oral and topical pharmacotherapy for neuropathic pain [3]. This analysis led to a strong GRADE recommendation for the use of tricyclic antidepressants (TCAs), serotonin-noradrenaline reuptake inhibitor (SNRI) antidepressants, pregabalin, gabapentin, and gabapentin/enacarbil XR and the proposal of these drugs as first-line treatments. In addition, the analysis provided a weak recommendation for the use of lidocaine patches, capsaicin patches, and tramadol, the proposal of these drugs as second-line treatments, a weak recommendation for the use of strong opioids (particularly oxycodone and morphine) and botulinum toxin A (BTX-A), and the proposal of these drugs as third-line treatments. Data for tapentadol and drug combinations were inconclusive. A weak recommendation was provided against cannabinoid use in neuropathic pain [3].

In the last few years, new medical treatments for neuropathic pain have been tested, including compounds acting on new pain targets [1].

We conducted a systematic review and meta-analysis of randomised controlled trials (RCTs) of all pharmacological treatments for neuropathic pain published in 2015 or later in order to analyse the efficacy and safety of drugs tested in the last five years and provide an update to the 2015 NeuPSIG meta-analysis.

## 2. Search Process

Systematic literature review was performed according to the Preferred Reporting Items for Systematic Reviews and Meta-Analyses (PRISMA) statements. We searched for relevant papers within PubMed, EMBASE, and the Cochrane Database of Systematic Reviews and considered studies published in peer-reviewed journals between January 2015 and July 2020. Search terms were related to pharmacological treatment of neuropathic pain. The primary search was supplemented by a secondary search using the bibliographies of retrieved articles. RCTs involving at least 10 patients were considered, and the search was limited to English language publications. Studies published only as abstracts were excluded. We checked ClinicalTrial.gov in order to include studies currently in progress. The target population was patients of any age with neuropathic pain according to the IASP definition (i.e., pain caused by a lesion or disease of the somatosensory nervous system) and related to different aetiologies. Mixed pain (e.g., cancer pain and low back pain) and conditions such as fibromyalgia and atypical facial pain were not included in the analysis.

**Figure 1:**
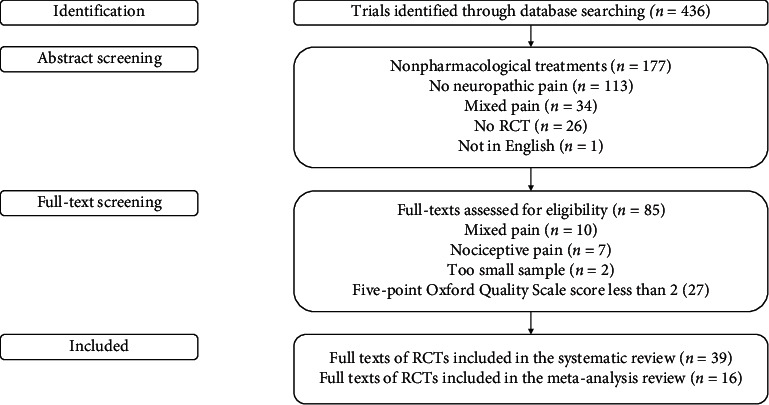
Flowchart of the search process.

The review process was performed independently by two reviewers (Figure 1). The authors independently assessed the quality of the individual trials during data extraction. Two independent authors assessed the studies in terms of methodological quality using the five-point Oxford Quality Scale [4]. A minimum score of 2 out of 5 (randomised and double-blind studies) was required for inclusion.

## 3. Statistics

The number needed to treat (NNT) for 50% pain intensity reduction (alternatively, 30% pain reduction) was considered as the primary effect measure in the meta-analysis. The 95% confidence interval (CI) for NNT values was calculated as the reciprocal value of the 95% CI for the absolute risk difference using normal approximation. Studies testing combination therapies were excluded from the meta-analysis. In dose-finding studies, data from subgroups treated with low doses were not included in the meta-analysis. Pooled NNT was calculated by considering a fixed-effects model based on the Mantel–Haenszel formula. The pooled variance of the studies, used to evaluate the confidence interval, was computed according to the Greenland–Robins formula. We assessed heterogeneity among studies by calculating the heterogeneity index *I*^2^ [5] on the basis of the pooled *χ*2. When the overall heterogeneity *I*^2^ exceeded a given threshold (30%), a random-effects model was considered, using the DerSimonian and Laird method for variance. Statistical analysis was performed using GraphPad Prism 8 software.

## 4. Results

We identified 436 RCTs in patients with neuropathic pain (Figure 1). After abstract screening, 85 full texts were assessed for eligibility. We excluded 17 studies that tested the effects of drugs in patients with mixed or nociceptive pain and two studies that involved a small number of patients (less than 10). We assessed the risk of bias in the remaining 66 trials through the five-point Oxford Quality Scale [4]. We excluded 27 trials with a score less than 2. Thirty-nine RCTs were included in the systematic review (Tables 1–7), and 16 RCTs that provided NNT values were included in the meta-analysis (Table 8).

### 4.1. Antidepressants

Five RCTs globally involving 486 patients tested the effects of antidepressants, including venlafaxine, duloxetine, amitriptyline, and imipramine, in patients with neuropathic pain related to different aetiologies (Table 1) [6–10]. In two RCTs, the effect of duloxetine 60 mg and amitriptyline 10 mg was compared to that of gabapentin 900 mg [6, 8]. In the study by Majdinasab et al., both duloxetine and gabapentin were effective in relieving pain in patients with painful diabetic neuropathy; though gabapentin showed an earlier effect, it had more side effects. Conversely, duloxetine had better medication compliance. At the end of a 6-week trial conducted by Brown et al., amitriptyline and gabapentin significantly decreased pain intensity scores without significant differences between the two drugs in terms of their effects on pain reduction and the frequency of adverse events. In the study by Farshchian et al., 156 patients with chemotherapy-induced peripheral neuropathy were randomly assigned to one of three pharmacotherapy groups: venlafaxine, duloxetine, or placebo. Neuropathic pain decreased significantly in both the venlafaxine and duloxetine groups as compared with placebo. Duloxetine was more effective than venlafaxine in reducing pain. No severe side effects were reported in the two groups.

**Figure 2:**
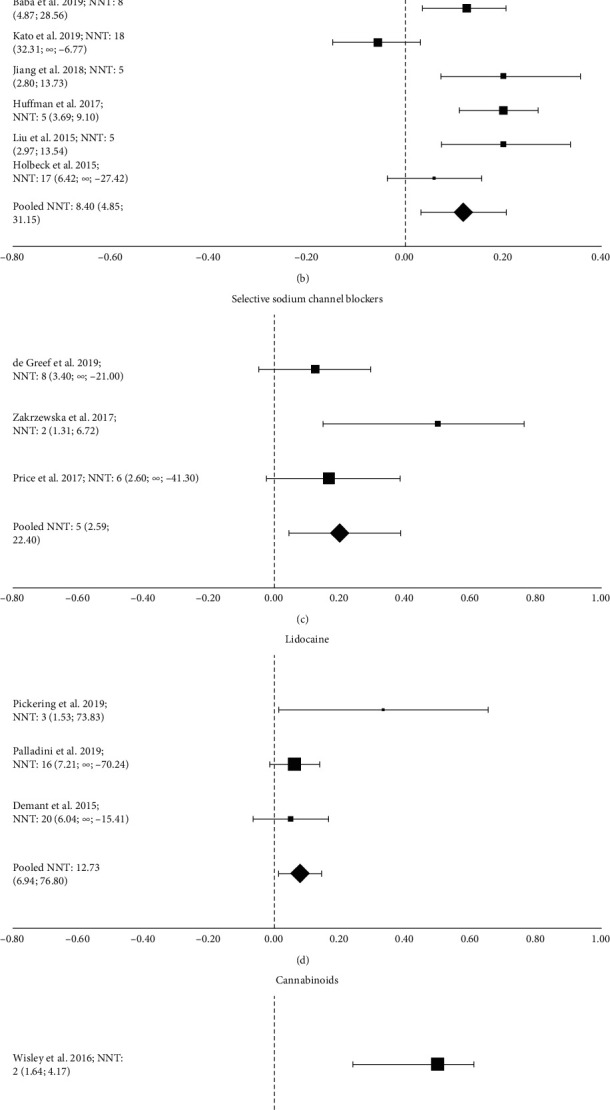
Forest plots of randomised controlled trials included in the metanalysis. Each value is expressed as mean NNT (95% CI).

Two studies involving 192 patients with neuropathic pain related to spinal cord injury and painful neuropathy were included in the metanalysis [9, 10]. The authors tested the effects of venlafaxine XR and imipramine in comparison with placebo. The effect of venlafaxine XR on neuropathic pain was similar to that of placebo [9]. Imipramine was tested both in monotherapy and in combination with pregabalin [10]. A 50% pain relief was reported by 20% of patients treated with imipramine. The combined NNT using a random-effects model was 12.41 (4.07; ∞; −11.83) (test for heterogeneity: *χ*^2^ = 2.1; d*f* = 1; *P*=0.15; *I*^2^ = 51.28) (Figure 2(a).

### 4.2. Gabapentinoids

Nine RCTs globally involving 3903 patients tested the effect of gabapentinoids on neuropathic pain related to different aetiologies, including diabetic polyneuropathy, postherpetic neuralgia, and radiotherapy-related neuropathic pain (Table 2) [10–18].

Four RCTs testing the effect of pregabalin [10, 12, 14, 15] and two RCTs testing the effect of mirogabalin [11, 13] were included in the meta-analysis. The combined NNT using a random-effects model was 8.40 (4.85; 31.15) (test for heterogeneity: *χ*^2^ = 24.2; d*f* = 5; *P*=0.0002; *I*^2^ = 79.32%) (Figure 2(b).

The most common side effects with pregabalin included dizziness, somnolence, oedema, and weight gain. In patients treated with mirogabalin, the most reported side effects were nasopharyngitis, somnolence, and dizziness (Table 2).

Two RCTs testing the effect of mirogabalin on central neuropathic pain and diabetic peripheral neuropathic pain are currently ongoing (ClinicalTrials.gov Identifier: NCT03901352; NCT04094662). The combination of gabapentin and trazodone is currently being tested in painful diabetic neuropathy (ClinicalTrials.gov Identifier: NCT03749642) (Lipone et al., 2020).

### 4.3. Lidocaine

Five RCTs globally involving 670 patients tested the effect of lidocaine in patients with localised peripheral neuropathic pain (Table 3) [19–23]. Three RCTs [19, 20, 23] testing the effect of the lidocaine patch 5% in localised peripheral neuropathic pain were included in the meta-analysis. The combined NNT using a fixed-effects model was 12.73 (6.94; 76.79) (test for heterogeneity: *χ*^2^ = 2.9; d*f* = 2; *P*=0.240; *I*^2^ = 29.86%) (Figure 2(d). The most common side effects included mild skin reactions (Table 4).

### 4.4. Opioids

Four RCTs globally involving 215 patients tested the effect of opioids in different neuropathic pain conditions, including postherpetic neuralgia, diabetic peripheral neuropathy, and central pain related to multiple sclerosis (Table 4) [24–27].

In the study by Gavin et al., the transdermal oxycodone patch was compared with placebo in patients with postherpetic neuralgia [24]. The authors found that the oxycodone patch did not significantly reduce pain, though patients reporting high levels of paraesthesia showed a trend toward improved pain reduction.

In the study by Simpson and Wlodarczyk, the buprenorphine patch up to 40 *μ*g/h was tested in comparison with placebo in patients with diabetic peripheral neuropathy [26]. A high proportion of patients did not complete the study due to adverse events, and the primary endpoint was not reached.

Two RCTs compared combination therapy with monotherapy [25, 27]. In the study by Rigo et al., the effect of oral methadone combined with oral ketamine was compared to that of monotherapy [25]. A significant pain improvement was observed in the ketamine alone group as compared with both the methadone and methadone/ketamine groups. In the study by Gilron et al., the nortriptyline-morphine combination was compared with each monotherapy [27]. The study showed superior efficacy of combination treatment as compared with monotherapy. The most frequent adverse events included constipation, dry mouth, and somnolence (Table 5).

### 4.5. Cannabinoids

Five RCTs globally involving 335 patients tested the effect of cannabinoids in patients with central neuropathic pain related to multiple sclerosis and spinal cord injury and in patients with pain related to diabetic peripheral neuropathy (Table 5) [28–32]. In the two RCTs involving patients with multiple sclerosis [28, 29], tetrahydrocannabinol (THC) did not significantly reduce pain in comparison with placebo.

The effect of nabilone 2 mg combined with gabapentin was compared with placebo in patients with central neuropathic pain related to multiple sclerosis [31]. Pain decrease was statistically greater in the nabilone vs. placebo study group. Nabilone was well tolerated, with dizziness and drowsiness being the most frequently reported side effects.

Two RCTs [30, 32] were included in the meta-analysis. The combined NNT using a random-effects model was 2.96 (1.78; 8.77) (test for heterogeneity: *χ*^2^ = 1.7; d*f* = 1; *P*=0.191; I^2^ = 41.41%) (Figure 2(e). The most frequently reported side effects included dizziness, somnolence, muscle weakness, paraesthesia, tremor, tinnitus, psychiatric/mood disorders, fatigue, and dry mouth (Table 6).

The effect of acutely administered cannabidiol/THC is currently being tested in HIV-related neuropathic pain (ClinicalTrials.gov Identifier: NCT03099005).

### 4.6. Sodium Channel Blockers

Four RCTs globally involving 319 patients tested the effect of selective sodium channel blockers in different neuropathic pain conditions, including trigeminal neuralgia, postherpetic neuralgia, and painful polyneuropathy (Table 6) [33–36]. A double-blind placebo-controlled randomised withdrawal phase IIa trial tested the effect of a new voltage- and frequency-dependent sodium channel blocker selective for the sodium channel 1.7 (Nav1.7) subtype in 67 patients with trigeminal neuralgia [35]. A 21-day open-label treatment period using BIIB074 150 mg three times/day was followed by randomisation into a double-blind 28-day treatment phase with either placebo or BIIB074 in only those patients who showed a successful treatment response within the final week. Although the primary endpoint of treatment failure was not significantly lower in the BIIB074 group as compared with the placebo group, significant treatment differences as compared with placebo were found in secondary endpoints, including time to treatment failure, number of paroxysms, and average daily pain score. A phase III trial is currently ongoing, though recruitment has not yet started (ClinicalTrials.gov Identifier: NCT03637387).

A novel selective Nav1.7 sodium channel blocker (PF-05089771) was also tested in 135 patients with diabetic peripheral neuropathy [34]. Although a trend toward a reduction in the weekly average pain score in the PF-05089771 treatment group was observed, this was not statistically significant when compared with placebo.

A novel topical sodium channel inhibitor (TV-45070) was compared with placebo in 70 patients with postherpetic neuralgia [36]. Although this study had a negative primary endpoint, it found a remarkable analgesic response in the subpopulation with the R1150W polymorphism (63% responders vs. 35% of wild-type carriers).

de Greef et al. tested the effect of lacosamide in 47 patients with Nav1.7-mutation-related small-fibre neuropathy [33]. In 58.3% of patients receiving lacosamide, mean average pain decreased by at least 1 point, compared to 21.7% in the placebo group.

The combined NNT using a random-effects model was 4.64 (2.59; 22.40) (test for heterogeneity: *χ*^2^ = 3.7; d*f* = 3; *P*=0.160; *I*^2^ = 45.46%) (Figure 2(c).

The effect of lacosamide on peripheral neuropathic pain with and without the irritable nociceptor phenotype is currently being tested (ClinicalTrials.gov Identifier: NCT03777956).

### 4.7. Other Drugs

Eight trials testing other drugs in a total of 865 patients were included in the systematic review (Table 7) [37–44].

A randomised double-blinded crossover placebo-controlled trial tested the efficacy and safety of 0.075% capsaicin lotion in 42 patients with painful diabetic neuropathy [37]. Intention-to-treat analysis showed no significant improvement in pain control with capsaicin lotion as compared with placebo for all pain measures.

The effect of ethosuximide was compared with placebo in patients with peripheral neuropathic pain [38]. The study was suspended during interim analysis due to the high number of adverse events in the active treatment group.

Oral mixed tocotrienols were tested in 300 patients with painful diabetic peripheral neuropathy [39]. Mixed tocotrienols 400 mg/day for 1 year did not reduce overall neuropathic symptoms. Tocotrienols were relatively well tolerated, with a safety profile comparable to that of placebo.

A phase IIa trial assessed the analgesic effect and safety of ASP8477, a fatty acid amide hydrolase inhibitor, in 132 patients with painful diabetic peripheral neuropathy and postherpetic neuralgia [40]. ASP8477 was well tolerated but did not demonstrate a significant treatment difference as compared with placebo.

Andresen et al. tested the effect of ultramicronised palmitoylethanolamide (PEA-um) in 73 patients with neuropathic pain related to spinal cord injury [41]. No difference in mean pain intensity between PEA-um and placebo was found. PEA-um was not associated with more adverse effects than placebo.

Two RCTs tested the effect of monoclonal antibodies in neuropathic pain treatment [42, 43]. In a phase II trial, fulranumab was compared with placebo in patients with postherpetic neuralgia and painful posttraumatic neuropathy [42]. Fulranumab did not significantly reduce pain in comparison with placebo. The most common adverse events included sinusitis, carpal tunnel syndrome, headache, and arthralgia. The study was prematurely suspended due to potential safety concerns with this drug class (rapidly progressive osteoarthritis).

Subcutaneous and intravenous tanezumab was compared with placebo in patients with diabetic peripheral neuropathy and postherpetic neuralgia [43]. Only diabetic patients reported greater mean pain reduction with tanezumab than with placebo. In patients with postherpetic neuralgia, only the highest tanezumab dose (200 *μ*g/kg) reduced pain, but treatment differences were not significant. The most common side effects included arthralgia and headache.

Topical diclofenac (1.5%) was compared with placebo in 28 patients with neuropathic pain [44]. After 2 weeks of topical application, subjects treated with diclofenac showed a significantly lower overall visual pain score compared with the placebo group.

Two randomised placebo-controlled trials testing the effect of EMA 401 (an oral angiotensin type-II antagonist) in postherpetic neuralgia (ClinicalTrials.gov Identifier: NCT03094195) and painful diabetic neuropathy (ClinicalTrials.gov Identifier: NCT03297294) were prematurely suspended due to preclinical toxicity data.

The CGRP receptor antibody, erenumab, is currently being compared with placebo in patients with trigeminal neuralgia (ClinicalTrials.gov Identifier: NCT04054024). Other drugs currently being tested in RCTs include N-desmethylclobazam in patients with peripheral neuropathic pain (ClinicalTrials.gov Identifier: NCT04480164), clonidine, and pentoxifylline in different neuropathic pain conditions (ClinicalTrials.gov Identifier: NCT03342950), ziconotide in patients with severe refractory neuropathic pain (ClinicalTrials.gov Identifier: NCT03942848), and brivaracetam in spinal cord injury (ClinicalTrials.gov Identifier: NCT04379011).

## 5. Discussion

This manuscript provides an update to the 2015 NeuPSIG meta-analysis on neuropathic pain treatment [3]. We analysed 39 RCTs in our systematic review and included 16 trials in our meta-analysis.

We found that the combined NNT was 12.41 (4.07; ∞; −11.83) for antidepressants (venlafaxine XR and imipramine), 8.40 (4.85; 31.15) for gabapentinoids (pregabalin and mirogabalin), 4.64 (2.59; 22.40) for selective sodium channel blockers, 12.73 (6.94; 76.79) for lidocaine, and 2.96 (1.78; 8.77) for cannabinoids (Figure 2). Admittedly, the low number of studies included in the meta-analysis may affect the external consistency of these NNT values.

We found poor trial outcomes even for first-line drugs such as antidepressants and pregabalin. These findings, consistent with those of the previous NeuPSIG metanalysis [3], may partly reflect the high placebo response and the lack of adequate diagnostic criteria for neuropathic pain. Another possible issue concerns the heterogeneity of patient phenotypes in clinical trials, which may underlie heterogeneous pathophysiological mechanisms and, thus, different drug responses. Recent clinical trials and post hoc analyses suggest that some drugs might be differentially effective in patients classified on the basis of their sensory phenotype [23, 45]. In the study by Demant et al., the sodium channel blocker oxcarbazepine relieved peripheral neuropathic pain more efficaciously in patients with the irritable nociceptor phenotype, in which upregulation of sodium channels in nociceptors has been proposed as the pain-generating mechanism [23]. These data highlight the need for better-targeted existing therapies and a mechanism-based personalised treatment of neuropathic pain.

Unexpectedly, in our meta-analysis, we found that antidepressants had a relatively high NNT, larger than that reported in the previous NeuPSIG meta-analysis. This finding, however, may merely reflect the inclusion of only two studies that tested the effect of venlafaxine XR and imipramine. We did not include any study on the efficacy of amitriptyline, the most widely used tricyclic antidepressant for neuropathic pain.

For pregabalin, we found a slightly larger combined NNT than that reported in the previous NeuPSIG metanalysis. The effect size of pregabalin and its clinical usefulness are currently sources of debate [46, 47]. Recent recommendations issued by the French chapter of the IASP and the French Society of Neurology [46] have proposed pregabalin as an alternative to gabapentin for second-line treatment, given that different studies [3, 48] have shown that gabapentin has a relatively large effect size and tolerability.

Our meta-analysis showed a large NNT for topical lidocaine (about 12). This finding is in line with previous large meta-analyses [3, 46]. However, due to its excellent safety profile, topical lidocaine is commonly recommended as a first- or second-line drug, particularly in patients with localised neuropathic pain [3, 46].

We found a large effect (NNT = 2.96) for cannabinoids. This finding is in contrast to the previous NeuPSIG recommendations, which provided a weak recommendation against the use of cannabinoids in patients with neuropathic pain [3]. Our finding on the efficacy of cannabinoids was probably affected by an overestimation of their effect due to the inclusion of only two studies. Hence, we believe that reliable evidence is still needed regarding the efficacy and safety of cannabinoids in patients with neuropathic pain.

In this meta-analysis, we have now included also selective Nav1.7 sodium channel blockers. We found a larger effect of new selective Nav1.7 sodium channel blockers than that of currently used neuropathic pain medications, including first-line drugs. Nav1.7 plays a key role in pain perception [49] and has a relatively specific expression in the peripheral nervous system, thus suggesting that selective Nav1.7 sodium channel blockers may have fewer side effects than currently available analgesics. Different studies are currently assessing the efficacy and safety of these new compounds in different neuropathic pain conditions. This research field is also supported by evidence of voltage-gated Na + channel variations in the pathogenesis of different neuropathic pain conditions [50–52]. The increasing availability of gene sequencing, combined with structural modelling and electrophysiological analysis of gene variants, may provide an opportunity to better target existing therapies. The anticonvulsant lacosamide, acting on Nav1.3, Nav1.7, and Nav1.8 channels, was tested in patients with Nav1.7-mutation-related small-fibre neuropathy, with promising findings in a subgroup of patients [33]. Labau and colleagues, using voltage-clamp recordings, found a preferential effect of lacosamide on Nav1.7 variants in patients who were responsive to lacosamide via a hyperpolarizing shift in the voltage dependence of both fast and slow inactivation and enhancement of use-dependent inhibition [53]. In addition, recent studies have shown that carbamazepine, at clinically achievable concentrations, acts via a new mode of action as an activation modulator of select mutant Nav1.7 channels [54, 55].

Another novel research field concerns the development of antibodies targeting the activity of human nerve growth factors. In a phase II trial in patients with postherpetic neuralgia and painful posttraumatic neuropathy [42], fulranumab did not significantly reduce pain in comparison with placebo. When subcutaneous and intravenous tanezumab was compared with placebo in patients with diabetic painful neuropathy and postherpetic neuralgia [43], pain relief was significantly higher with tanezumab than with placebo in only diabetic patients.

Among the new emerging drugs, ASP8477, a selective fatty acid amide hydrolase inhibitor, is under development. This drug was recently tested in patients with peripheral neuropathic pain and showed a good safety profile, though no significant pain relief as compared with placebo was found [40].

## 6. Conclusions

Our systematic review and meta-analysis, which included data from randomised controlled trials in patients with neuropathic pain, extend previous knowledge by demonstrating small effect sizes and/or large NNTs for all compounds used to treat neuropathic pain [2]. The modest efficacy of available treatments suggests the need for novel drug options. Drugs acting on new pain targets, including sodium channel isoform-specific antagonists and monoclonal antibodies, are currently under development. In particular, the new selective Nav1.7 sodium channel blockers might be an effective treatment option for a selected population [49, 50].

## Figures and Tables

**Table 1:** Randomized, double-blind, controlled trials testing the effect of antidepressants.

Study	Active drug	Control	Sample size	NP condition	Outcome measure	Pain outcome	Adverse events
Majdinasab et al. [6]	Duloxetine 60 mg	Gabapentin 900 mg	104	DPN	0–100 VAS	Positive with both drugs	Anxiety (2%) and sleeplessness (2%)
Farshchian et al. [7]	Venlafaxine 37.5 mg; duloxetine 30 mg	Placebo	156	CIPN	0–3 neuropathic pain grade	Positive	Venlafaxine: nausea (12%), constipation (7%), and insomnia (7%); duloxetine: dizziness (11%), fatigue (10%), and headache (4%)
Brown et al. [8]	Amitriptyline 10 mg	Gabapentin 900 mg	34	Different NP conditions	0–10 CAS score	Positive with both drugs	Not reported
Richards et al. [9]	Venlafaxine XR 75–225 mg	Placebo	123	SCI	0–10 NRS	Negative	Not reported
Holbech et al. [10]	Imipramine 75 mg	Placebo	69	Painful polyneuropathy	0–10 NRS	Positive	Dizziness (10%), sweating (20%) dry mouth (22%), and paraesthesia (10%)

NP: neuropathic pain; DPN: diabetic painful neuropathy; CIPN: chemotherapy-induced peripheral neuropathy; SCI: spinal cord injury; NRS: numerical rating scale; CAS: colour analogue scale; VAS: visual analogue scale.

**Table 2:** Randomized, double-blind, controlled trials testing the effect of gabapentinoids.

Study	Active drug	Control	Sample size	NP condition	Outcome measure	Pain outcome	Adverse events (>10%)
Baba et al. [11]	Mirogabalin 15/20/30 mg	Placebo	834	DPN	0–10 NRS	Positive	Nasopharyngitis (16.4%), somnolence (14.5%), and dizziness (18%)
Jiang et al. [12]	Pregabalin 300–600 mg	Placebo	128	Radiotherapy-induced NP	0–10 NRS	Positive	Dizziness (18.8%) and somnolence (20.3%)
Kato et al. [13]	Mirogabalin 15/20/30 mg	Placebo	765	PHN	0–10 NRS	Positive	Somnolence (23.9%), nasopharyngitis (12.9), and dizziness (15.5%)
Huffman et al. [14]	Pregabalin CR 82.5–660 mg	Placebo	413	PHN	NPS	Positive	Dizziness (17.1%) and somnolence (11.4%)
Liu et al. [15]	Pregabalin 300 mg	Placebo	220	PHN	0–10 NRS	Positive	Dizziness (24.3%)
Merante et al. [16]	Mirogabalin 15/20/30 mg	Placebo	452	DPN	PGIC, BPI	Positive	Dizziness (15.8%) and somnolence (12.3%)
Raskin et al. [17]	Pregabalin 150–300 mg	Placebo	301	DPN	0–10 NRS	Negative	Dizziness (10.3)
Freeman et al. [18]	Gabapentin 1800 mg	Placebo	721	PHN	0–100 NRS	Positive	Not reported
Holbech et al. [10]	Pregabalin 300 mg	Placebo	69	Painful polyneuropathy	0–10 NRS	Positive	Dizziness (16%), oedema (16%), and weight gain (14%)

NP: neuropathic pain; DPN: diabetic painful neuropathy; PHN: postherpetic neuralgia; NRS: numerical rating scale; NPS: neuropathic pain scale; PGIC: patient global impression of change; BPI: brief pain inventory.

**Table 3:** Randomized, double-blind, controlled trials testing the effect of lidocaine.

Study	Active drug	Control	Sample size	NP condition	Outcome measure	Pain outcome	Adverse events
Palladini et al. [19]	Lidocaine 5% 12 h/day	Placebo	363	Localized NP	0–10 NRS	Negative	Pain (7.3%), headache (5.6%), gastroenteritis (2.8%), and application site pruritus (2.8%)
Pickering et al. [20]	Lidocaine 5% 12 h/day	Placebo	36	Localized NP	0–10 NRS	Positive	Skin and subcutaneous tissue disorders (25.0%)
Kim et al. [21]	Lidocaine IV (3 mg/kg)	Placebo	42	PHN or CPRS	0–10 NRS	Positive	Chest discomfort in one patient
Liu et al. [22]	Lidocaine IV (5 mg/kg)	Placebo	183	PHN	VAS	Negative	Dizziness (21.1%), dry mouth (15.6%), headache (6.7%), and drowsiness (5.6%)
Demant et al. [23]	Lidocaine 5% 12 h/day	Placebo	46	Localized NP	0–10 NRS	Positive	Mild skin reaction (21%)

NP: neuropathic pain; NRS: numerical rating scale; PHN: postherpetic neuralgia; CPRS: complex regional pain syndrome; VAS: visual analogue scale.

**Table 4:** Randomized, double-blind, controlled trials testing the effect of opioids.

Study	Active drug	Control	Sample size	NP condition	Outcome measure	Pain outcome	Adverse events
Gavin et al. [24]	Oxycodone patch 23.6 mg/72 hours	Placebo	28	PHN	0–10 NRS	Negative	Application site irritation (18.5%), infections (7.4%), respiratory disorders (7.4%), gastrointestinal disorders (3.7%), musculoskeletal, and connective tissue disorders (3.7%)
Rigo et al. [25]	Methadone 3 mg; ketamine 30 mg	Methadone 3 mg or ketamine 30 mg	42	Refractory NP	VAS	Negative*∗*	Somnolence (46%), nausea (23%), vomiting (15%), and constipation (8%)
Simpson and Wlodarczyk [26]	Buprenorphine patch <40 mg/h	Placebo	93	DPN	0–10 NRS	Negative	Nausea (43.0%) and constipation (31.2%)
Gilron et al. [27]	Morphine 10 mg; nortriptyline 10 mg	Nortriptyline 10 mg or morphine 10 mg	52	Peripheral NP	0–10 NRS	Positive	Dry mouth (57.5%), constipation (42.5%), and somnolence (20%)

NP: neuropathic pain; DPN: diabetic painful neuropathy; PHN: postherpetic neuralgia; NRS: numerical rating scale; VAS: visual analog scale. ^*∗*^A significant pain relief was observed in the ketamine alone group compared with both the methadone and methadone/ketamine groups.

**Table 5:** Randomized, double-blind, controlled trials testing the effect of cannabinoids.

Study	Active drug	Control	Sample size	NP condition	Outcome measure	Pain outcome	Adverse events (>10%)
Schimrigk et al. [28]	Dronabinol 7.5–15.0 mg	Placebo	238	MS	0–10 NRS	Negative	Dizziness (17.4%)
van Amerongen et al. [29]	D9-THC 16 mg	Placebo	24	MS	0–10 NRS	Negative	Dizziness (58.3%), headache (50%), muscular weakness (33.3%), somnolence (25%), paraesthesia (16.7%), tremor (16.7%), tinnitus (16.7%), psychiatric/mood (33.3%), fatigue (16.7%), and dry mouth (16.7%)
Wilsey et al. [30]	D9-THC 2.9–6.7%	Placebo	42	SCI	0–10 NRS	Positive	Not reported
Turcotte et al. [31]	Nabilone 2 mg; gabapentin ≥1.800 mg	Placebo	15	MS-related NP	VAS	Positive	Dizziness (62.5%), drowsiness (50%), and dry mouth (50%)
Wallace et al. [32]	D9-THC 1/4/7%	Placebo	16	DPN	VAS	Positive	Euphoria (100%) and somnolence (73.3%)

NP: neuropathic pain; MS: multiple sclerosis; DPN: diabetic painful neuropathy; NRS: numerical rating scale; SCI: spinal cord injury; VAS: visual analog scale.

**Table 6:** Randomized, double-blind, controlled trials testing the effect of sodium channel blockers.

Reference	Active drug	Control	Sample size	NP condition	Outcome measure	Pain outcome	Adverse events
de Greef et al. [33]	Lacosamide 400 mg	Placebo	47	Nav1.7-related SFN	0–10 NRS	Positive	Dizziness (41.7), nausea (25%), headache (25%), fatigue (20.8%), tremor (20.8%), somnolence (16.7%), epigastric discomfort (16.7%), memory impairment (12.5%), and pruritus (12.5%)
Mc Donnell et al. [34]	PF-05089771 300 mg	Placebo	135	DPN	0–10 NRS	Negative	Constipation (5%), back pain (1%) headache (1%), and pollakiuria (1%)
Zakrzewska et al. [35]	BIIB074 450 mg	Placebo	67	TN	Number of treatment failures	Negative	Headache (19%), dizziness (9%), dyspepsia (6%), diarrhoea (6%), abdominal pain (6%), and fatigue (6%)
Price et al. [36]	TV-45070 ointment	Placebo	70	PHN	Mean daily pain score	Negative	Application site pain (15.9%), application site pruritus (12.7%), and infections (17.5%)

NP: neuropathic pain; SFN: small-fibre neuropathy; DPN: diabetic painful neuropathy; TN: trigeminal neuralgia; PHN: postherpetic neuralgia; NRS: numerical rating scale.

**Table 7:** Randomized, double-blind, controlled trials testing the effect of other drugs.

Study	Active drug	Control	Sample size	NP condition	Outcome measure	Pain outcome	Adverse events
Kulkantrakorn et al. [37]	Capsaicin 0.075%	Placebo	42	DPN	VAS	Negative	Skin reaction (50%), burning sensation (41.7%), and erythema (11.1%)
Kerckhove et al. [38]	Ethosuximide 1500 mg	Placebo	114	Peripheral NP	0–10 NRS	Negative	Dyspepsia (39%), headache (32%), and dizziness (20%).
Hor et al. [39]	Tocotrienols 400 mg	Placebo	300	DPN	TSS	Negative	Not reported
Bradford et al. [40]	ASP8477 20–60 mg	Placebo	132	DPN and PHN	0–10 NRS	Negative	Allergic dermatitis (2.7%), increased appetite (2.7%), and musculoskeletal stiffness (2.7%)
Andresen et al. [41]	PEA-um 600 mg	Placebo	73	SCI	0–10 NRS	Negative	Urinary tract infection (1%), paralytic ileus (1%), cholecystolithiasis, and fungus infection (1%)
Wang et al. [42]	Fulranumab 1-3-10 *μ*g	Placebo		PHN and PTN	0–10 NRS	Negative	PHN: arthralgia (21%), osteoarthritis (21%), back pain (11%), oedema (11%), diarrhoea (11%), anaemia (11%), influenza (11%), and urinary tract infection (11%); PTN: sinusitis (17%), headache (13%), and carpal tunnel syndrome (13%)
Bramson et al. [43]	Tanezumab sc 20 *μ*g	Placebo	73	DPN	0–10 NRS	Positive	Arthralgia (18.4%) and pain in the extremity (10.5%)
Bramson et al. [43]	Tanezumab iv 50–200 *μ*g/kg	Placebo	96	PHN	0–10 NRS	Negative	Headache (12.5%)
Ahmed et al. [44]	Topical diclofenac 1.5%	Placebo	35	Different NP conditions	VAS	Positive	Not reported

NP: neuropathic pain; DPN: diabetic painful neuropathy; PHN: postherpetic neuralgia; NRS: numerical rating scale; SCI: spinal cord injury; VAS: visual analog scale. PTN: posttraumatic neuropathy.

**Table 8:** Number needed to treat.

Study and drug classes	Drug	NNT	ARR
Antidepressants			
Richards [9]	Venlafaxine XR 225 mg	38 (5.34; ∞; −4.15)	0.027 (−0.24; 0.19)
Holbech [10]	Imipramine 75 mg	7 (4,00; 32.51)	0.14 (0.03; 0.25)
Pooled		13 (4.07; ∞; −11.83)	0.08 (−0.09; 0.25)

Gabapentinoids			
Baba [11]	Mirogabalin 30 mg	8 (4.87; 28.56)	0.12 (0.04; 0.20)
Kato [13]	Mirogabalin 30 mg	18 (32,31; ∞; −6.77)	0.06 (−0.15; 0.03)
Jiang [12]	Pregabalin 300–600	5 (2.80; 13.73)	0.22 (0.09; 0.35)
Huffman [14]	Pregabalin CR 82.5–660 mg	5 (3.69; 9.10)	0.19 (0.12; 0.27)
Liu [15]	Pregabalin 300 mg	5 (2.97; 13.54)	0.21 (0.08; 0.34)
Holbech [10]	Pregabalin 300 mg	17 (6.42; ∞; −27.42)	0.06 (−0.04; 0.16)
Pooled		9 (4.85; 31.15)	0.12 (0.03; 0.21)

Cannabinoids			
Wilsey [30]	D9-THC 2.9–6.7%	2 (1.64–4.17)	0.43 (0.24; 0.61)
Wallace [32]	D9-THC 1-4-7%	5 (2.03; ∞; −8.53)	0.19 (−0.12; 0.49)
Pooled		3 (1.78; 8.77)	0.34 (0.11; 0.563)

Pickering [20]	Lidocaine patch 5%	3 (1.53; 73.83)	0.33 (0.01; 0.65)
Palladini [19]	Lidocaine patch 5%	16 (7.21; ∞; −70.24)	0.06 (−0.01; 0.14)
Demant [23]	Lidocaine patch 5%	20 (6.06; −15.41)	0.05 (−0.07; 0.17)
Pooled		13 (6.94; 76.80)	0.08 (−0.01; 0.16)

Sodium channel blockers			
de Greef [33]	Lacosamide 400 mg	8 (3,40; ∞; −21,00)	0.12 (−0.05; 0.29)
Zakrzewska [35]	BIIB074 450 mg	2 (1.31; 6.72)	0.46 (0.15; 0.77)
Price [36]	TV-45070	6 (2.60; ∞; −41.3)	0.18 (−0.02; 0.38)
Pooled		5 (2.59; 22.40)	0.22 (0.05; 0.39)

## Data Availability

All data generated or analysed during this study are included in this published article.
